# The case for low carbohydrate diets in diabetes management

**DOI:** 10.1186/1743-7075-2-16

**Published:** 2005-07-14

**Authors:** Surender K Arora, Samy I McFarlane

**Affiliations:** 1 Division of Endocrinology, Diabetes and Hypertension, SUNY Downstate Medical Center, and Kings County Hospital Center, Brooklyn, NY 11203 NY 11203, USA

## Abstract

A low fat, high carbohydrate diet in combination with regular exercise is the traditional recommendation for treating diabetes. Compliance with these lifestyle modifications is less than satisfactory, however, and a high carbohydrate diet raises postprandial plasma glucose and insulin secretion, thereby increasing risk of CVD, hypertension, dyslipidemia, obesity and diabetes. Moreover, the current epidemic of diabetes and obesity has been, over the past three decades, accompanied by a significant decrease in fat consumption and an increase in carbohydrate consumption. This apparent failure of the traditional diet, from a public health point of view, indicates that alternative dietary approaches are needed. Because carbohydrate is the major secretagogue of insulin, some form of carbohydrate restriction is a prima facie candidate for dietary control of diabetes. Evidence from various randomized controlled trials in recent years has convinced us that such diets are safe and effective, at least in short-term. These data show low carbohydrate diets to be comparable or better than traditional low fat high carbohydrate diets for weight reduction, improvement in the dyslipidemia of diabetes and metabolic syndrome as well as control of blood pressure, postprandial glycemia and insulin secretion. Furthermore, the ability of low carbohydrate diets to reduce triglycerides and to increase HDL is of particular importance. Resistance to such strategies has been due, in part, to equating it with the popular Atkins diet. However, there are many variations and room for individual physician planning. Some form of low carbohydrate diet, in combination with exercise, is a viable option for patients with diabetes. However, the extreme reduction of carbohydrate of popular diets (<30 g/day) cannot be recommended for a diabetic population at this time without further study. On the other hand, the dire objections continually raised in the literature appear to have very little scientific basis. Whereas it is traditional to say that more work needs to be done, the same is true of the assumed standard low fat diets which have an ambiguous record at best. We see current trends in the national dietary recommendations as a positive sign and an appropriate move in the right direction.

## The case for low carbohydrate diets in diabetes management

The epidemic of obesity and diabetes in our society over the past three decades has been accompanied by a decline in fat consumption and an apparent attempt to adopt the traditionally recommended low fat diet [1,2]. According to the USDA Continuing Survey of Food Intakes by Individuals (CSFII) [2], the absolute amount of fat and saturated fat consumed has decreased during the obesity epidemic although there is slight increase for women from 1994 to 1995. This apparent failure of low fat diets in curbing the obesity pandemic calls into question the effectiveness and long-term usefulness of such dietary recommendation and has led to renewed interest in alternative dietary interventions, notably those recommending reduced carbohydrate intake. Low fat diets are generally associated with high carbohydrate intake which in turn is associated with several metabolic abnormalities [3,4]. These metabolic abnormalities are more pronounced in the diabetic population, leading to worsening glycemic control, dyslipidemia and increased inflammation to name a few. In this review, we discuss the current evidence for a low carbohydrate diet versus a low fat diet in the management of people with diabetes, highlighting the potential role of low carbohydrate diet in ameliorating various metabolic abnormalities associated with diabetes.

## Carbohydrate restriction

It is important to understand that there is no clear cut definition of a low carbohydrate diet in the literature. Various popular versions recommend carbohydrates intake < 20% of caloric intake with absolute amounts < 50–60 gm/day, sometime as low as ≤ 20–30 gm/day at least for short periods. We distinguish between moderate but significant reduction in carbohydrates **(LoCHO diet) **and very low carbohydrate ketogenic diets **(VLCKD) **with extreme reductions (< 20 or 30 g/day) as in the early phase of the various popular diets [5-7]. The caloric deficit due to carbohydrate restriction may be balanced with increased intake of proteins and fats although the distribution is not always clear in the application of popular diets and, in at least two studies, no increase in dietary intake of proteins or fats was observed presumably due to effect of LoCHO diet on appetite and satiety [9,12] It is interesting that despite advocating *ad libitum *fat and protein intake, a LoCHO diet may be hypocaloric either by design or by spontaneous reduction of intake [8-12].

## Low carbohydrate diets and weight loss

Data from various studies demonstrate that even a modest loss of 5–10% of initial body weight may significantly improve glycemic control, hyperinsulinemia and other metabolic abnormalities [13,14]. In the Diabetes Prevention Program **(DPP) **and the Finnish trials, lifestyle intervention including modest weight loss was effective in preventing the development of diabetes in a high risk population [15,16]. Weight control, *per se*, is thus a critical component for achieving glycemic control, improving insulin resistance and modifying CVD risk in patients with diabetes and insulin resistance as well as for diabetes prevention [14,17].

Traditionally, increased fat intake has been considered as the main cause for excess energy intake and obesity but the trends in food intake during the obesity epidemic do not support this notion [2,18]. While fat intake has decreased, carbohydrate intake has increased simultaneously. This rise in dietary intake of carbohydrates, and especially highly refined carbohydrate, is a likely culprit in promoting weight gain and obesity [19].

Weight change is governed by two factors: caloric balance and macronutrient composition. The first has general agreement and the expectation is that any hypocaloric diet, should be effective in achieving weight loss [20]. As noted above, LoCHO or VLCKD are frequently intentionally or spontaneously low calorie. The second consideration, macronutrient composition, is more controversial. Comparisons of isocaloric diets of different macronutrient composition frequently show no difference in effectiveness but there are several examples where distinct advantages accrue to one of the diets, usually the low carbohydrate arm [21-25].

In a recent study [21], for example, significantly greater weight loss was demonstrated with low carbohydrate intervention (< 10% calories from carbohydrates) despite higher caloric intake (1855 kcal/day) compared to high carbohydrate (60% calories from carbohydrates) with lower caloric intake (1562 kcal/day). There are several other reports indicating metabolic advantage in low carbohydrate diets over short term (3–6 months) [8,10,11,26-28]. Significant reductions in fat mass including truncal fat, which is a marker for visceral obesity, have been demonstrated in many studies [9,11,26,29]. A recent report [30] indicates that the effect will be seen primarily in subjects with insulin resistance. The association of insulin resistance with diabetes makes this of great importance.

Although the exact mechanism for this metabolic advantage is unknown, it is has been attributed to greater thermogenic effect of proteins in the face of increased demand for gluconeogenesis, increased futile cycling and increase in mitochondrial uncoupling [21,22,24]. Despite evidence suggesting more weight loss with isocaloric low carbohydrate diets, the issue of metabolic inefficiency with low carbohydrate dietary interventions is controversial and still not universally accepted.

The data for long term effectiveness of LoCHO diet is limited to studies with small sample size, poor adherence to dietary assignment in all dietary groups and inability to control the dietary carbohydrate amount over longer duration, making it difficult to demonstrate an appreciable difference between the dietary interventions. It is important to stress, however, that the same disclaimer must be made for low fat diets. Whereas calorie reduction by any means will lead to weight loss, the only comparisons of low fat diets are exactly the ones with low carbohydrate diets and few researchers would maintain that low fat diets have great compliance or long term effects that can be attributed to the particular regimen [31]. Two of the low carbohydrate-low fat comparisons were continued for 1 year [8,27]. It is frequently cited that the difference in weight loss between the LoCHO diet and low fat diet was not statistically significant after one year but it should be pointed out that in these studies, participants had the freedom to increase the carbohydrate content of the diet over longer duration and it is reasonable to say that as carbohydrate is added back to the diet, its effectiveness wanes. For example, in the study by Foster et al. [8], there was no significant difference in the urinary ketone levels between the two study groups after 3 months, suggesting inadequate carbohydrate restriction during the later part of the study which would contribute to the similarity in various parameters between the groups. In addition, the authors of these studies included subjects who had dropped out of the study. This method, justified under the name *"intention to treat analysis" *obscures the information in the study and has the effect of making the more effective diet look worse. In another recent study [32] comparing the effects of four popular diets including LoCHO diet and low fat diet, there were no significant differences in weight loss in the different groups at the end of 1 year. However, this study also had the shortcomings of the above studies, including small sample size (40 subjects in each group) and poor adherence in all the groups (30–60% dropouts). The LoCHO diet group also failed to reach carbohydrate reduction goal with carbohydrate intake of 190 gm/day at 6 months and 12 months as compared to baseline of 239 gm/day. Hence, it is not surprising that weight loss was not significantly different in LoCHO diet group. What is encouraging is that despite such marginal carbohydrate restriction in the LoCHO group, this group was able to achieve a modest weight loss that was comparable to the other diet groups, while maintaining a greater improvement in lipid profile suggesting that even minimal carbohydrate restriction may have beneficial effects in term of weight loss and might be offered to those at high risk who fail to lose weight with traditional low fat diet.

## Low carbohydrate diet and glycemic control

Diets containing 50–60% calories from carbohydrates have been the standard recommendation for patients with type 2 diabetes and metabolic syndrome [33-35]. However, evidence from several epidemiological studies such as the Nurses Health Study [36] and Health Professional Follow-Up Study [37] has linked dietary carbohydrate intake (measured as glycemic load) with risk of type 2 diabetes and CVD. In the Framingham Offspring Study [38], high glycemic index and glycemic loads were positively associated with metabolic syndrome. Prospective cohort studies have also linked carbohydrates with development of diabetes [39,40]. Compelling evidence from clinical and metabolic studies demonstrate worsening of glycemic control and dyslipidemia in diabetics with high carbohydrate diet [3,4,41,42] whereas low carbohydrate diet may reverse these serious metabolic abnormalities [10,27,43-46]

Carbohydrates are the major insulin secretagogues [47] and glycemic control in diabetic subjects is greatly influenced by dietary carbohydrate content. In fact, before the discovery of insulin, dietary carbohydrate restriction was the recommended treatment for diabetes management [48]. While subjects with type 1 diabetes are generally counseled to count dietary carbohydrates and adjust insulin dose accordingly [35], the concept of carbohydrate restriction in type 2 diabetes is not adequately emphasized. High carbohydrate intake is generally recommended, resulting in suboptimal glycemic control and lipoprotein profile, gradually increasing insulin and/or oral hypoglycemic medication requirement and weight gain. On the other hand, restriction of dietary carbohydrates is associated with improvement in glycemic control and other parameters of insulin resistance including body mass and lipid profile[8-10,43,45].

In the analysis of effects of macronutrient composition of diet on glycemic control, it is essential to differentiate the effect of carbohydrate restriction from that of weight loss so as to determine if the diet has beneficial effect on glycemic control independent of weight loss. This has been clarified by short term study in weight stable diabetic patients where carbohydrate restriction resulted in significant decrease (8.1% to 7.3%, p < 0.05) in glycosylated hemoglobin (HbA1c) compared to a high carbohydrate control diet [46]. In another study by the same group [45] in 8 diabetic men in a randomized 5-week cross over design with a 5-week wash out period, even larger beneficial effects on glycemic control were observed with low carbohydrate intervention (carbohydrate 20%, protein 30% and fat 50%) compared to control diet (carbohydrate 55%, protein 15% and fat 30%). The low carbohydrate diet had lower HbA1c (7.6 % ± 0.3), glucose levels and insulin levels compared to high carbohydrate group (HbA1c 9.8 % ± 0.5) despite similar weight loss with both diets. These data demonstrate that the benefits of low carbohydrate diet on glycemic control are independent of weight loss and are primarily due to carbohydrate reduction.

In a recent study [43] on obese diabetic subjects, a LoCHO diet (20% carbohydrates) was associated with a significant reduction in body weight, BMI, fasting blood glucose and HbA1C at 6 months compared to the high carbohydrate group (60% carbohydrates). Significant decreases in insulin and hypoglycemic medication requirement were also observed in the low carbohydrate diet group. Similar improvements in glycemic control were also reported by Boden et al. [9]. The study of Samaha et al. [10] also reported a decrease in mean fasting plasma glucose (FPG) levels in diabetic subjects with low carbohydrate diet compared to low fat diet group. The decrease in FPG correlated with the weight loss in this study though the one year data did not show any significant difference, likely due to inability to achieve target carbohydrate intake in the LoCHO diet group and to the significant number of dropouts affecting the power of the study to measure a statistically significant difference.

To summarize, the effect of LoCHO diet on glycemic control was significantly greater and occurred independent of weight loss in those studies that were able to achieve and maintain adequate carbohydrate restriction. In other studies, the effect on glycemic control was modest and proportional to the weight loss, and at least comparable to that seen with low fat diet.

In conclusion, low carbohydrate diet is associated with significant improvement in glycemic control and has the potential for reduction in need for exogenous insulin or oral hypoglycemic medications. Increased monounsaturated fatty acid **(MUFA) **intake and reduction of saturated fat intake may further improve the insulin sensitivity and glycemic control with low carbohydrate diet.

## Low carbohydrate diet and postprandial hyperglycemia

Postprandial hyperglycemia is a risk factor for CVD, particularly in diabetic patients [49-51]. Many studies including the Nurses Health Study [36] have suggested a link between dietary carbohydrates (measured in terms of glycemic load) and CVD risk. Furthermore, control of postprandial hyperglycemia has been shown to provide cardiovascular benefits, and to contribute to the overall decrease of hemoglobin A1c, something that has been clearly shown to reduce microvascular disease in both type 1 and type 2 diabetes [52,53]. Dietary carbohydrates are the major determinants of postprandial glucose levels [17,47,54] and LoCHO diets have been reported to lower postprandial glucose levels directly and indirectly by way of weight loss and may have beneficial effects on CVD risk factors [4,14]. Significant reductions in postprandial plasma glucose and plasma insulin levels with LoCHO diet have been demonstrated in many studies [4,9,42,55]. Furthermore, control of postprandial hyperglycemia with acarbose, an α-glucosidase inhibitor, has been demonstrated to significantly decrease the risk of diabetes in patients with impaired glucose tolerance [56,57].

## Low Carbohydrate diet and Dyslipidemia

Type 2 diabetes and metabolic syndrome are commonly associated with atherogenic dyslipidemia, characterized by elevated triglycerides (TG) levels and low HDL levels [17,58]. Additionally, qualitative changes in LDL cholesterol may be present in the form of small, dense LDL particles which are more atherogenic and may be associated with higher risk of CVD [58-60]. Evidence from various studies has confirmed that LDL, HDL and triglycerides are independent predictors of CVD [17,61-63]. Since nearly 75 % of diabetics die of heart disease, control of diabetic dyslipidemia is an important strategy in the primary prevention of CVD and a low fat high carbohydrate diet has been the standard recommendation from various health organizations to achieve this target [33-35]. A mounting body of evidence however, has demonstrated that the traditional low fat high carbohydrate diet is associated with elevated triglyceride and low HDL cholesterol levels and may worsen the dyslipidemia of type 2 diabetes and metabolic syndrome [3,10,25,27,28]. Reduction in dietary intake of fat is frequently associated with increased intake of carbohydrates and leads to carbohydrate induced hypertriglyceridemia (HPTG) [64-69]. This phenomenon has been observed in subjects consuming high carbohydrate low fat diets for as few as 5 days, with replacement of as little as 10% fat with carbohydrate and with dietary fat intake of as much as 30% of energy [64,65]. Decreasing fat without increasing carbohydrate does not appear to elevate triglycerides, thereby suggesting that addition of carbohydrates and not reduction in fat is responsible for this HPTG seen with high carbohydrate low fat diets. Though the exact mechanism for carbohydrate induced HPTG has not been clearly elucidated, both increase in TG synthesis and decrease in fractional TG clearance have been demonstrated [64-66] with a possible contribution from increased hepatic *de novo *fatty acid synthesis [64,67]. A number of factors influence the occurrence of carbohydrate induced HPTG and these include high BMI (>28 kg/m^2^), insulin resistance, post menopausal state, and genetic factors [64,65]. Diabetic, insulin resistant and obese subjects are thus at even higher risk. In addition, type and form of carbohydrates, particularly high sugar/starch ratio also contribute to carbohydrate induced HPTG [64]. Conversely, LoCHO diets have been consistently demonstrated to lower triglycerides and increase HDL [8-11,26-28]. Even the studies which failed to show significant differences in weight loss between LoCHO diet and low fat diets after one year [8,27] demonstrated significant reduction in TG and an increase in HDL with the LoCHO diet despite inability of subjects to achieve target carbohydrate intake. This result suggests that the improvement in TG is not only independent of weight loss but, again, even modest reduction in carbohydrate intake may have significant benefits on lipids. Significant clinical implications comes from the VA-HIT study [61], where a modest reduction in TG and elevation of HDL cholesterol were associated with notable improvement in CVD mortality.

Though weight loss *per se*, in combination with increased physical activity, is usually associated with an increase in HDL cholesterol and decreases in triglyceride and LDL cholesterol concentration, the beneficial effects on lipids of the caloric reduction in LoCHO diets appear to be secondary or additive to carbohydrate restriction and are seen even after adjusting for amount of weight loss[27].

A low fat diet, in the presence of weight loss is effective in lowering serum LDL cholesterol. On the other hand, such a regimen decreases HDL cholesterol without a significant increase in LDL size to less atherogenic form [70]. Emerging evidence suggests that LoCHO diets may actually have beneficial effects on LDL cholesterol by decreasing LDL particle concentration and increasing LDL size to less atherogenic form [25,28,70-73].

In summary, a low carbohydrate diet may be more effective than a low fat diet at improving the characteristic dyslipidemia associated with diabetes, namely high TG, low HDL and increased small dense LDL particles [70].

## Low carbohydrate diet and insulin resistance

LoCHO diets have been reported to have beneficial effect on hyperinsulinemia seen in type 2 diabetes and insulin resistance [8-10,45]. The data is, however, limited by few studies with small number of diabetic subjects and differences in method of measuring insulin sensitivity in various studies. Boden *et al*. demonstrated significant improvement in insulin sensitivity, up to 75%, with a low carbohydrate diet as measured by euglycemic hyperinsulinemic clamp method [9]. In another study [29], significant decreases in insulin to glucose ratio were seen in the LoCHO group suggesting improved insulin sensitivity, especially in subjects with insulin resistance and higher baseline insulin levels. Similar improvement in insulin sensitivity was reported by Gannon, *et al *[45]. In the studies by Samaha *et al*. [10] and by Foster *et al *[8], carbohydrate restriction was associated with a significant increase in insulin sensitivity at 6 months (measured only in non-diabetic subjects) although the difference between the low fat and low carbohydrate groups was not statistically significant at 1 year [27]. Notably, again, these studies allowed increasing carbohydrate in the LoCHO group with time thereby reducing the effectiveness of this group. Reduction in visceral obesity and omental fat may be important since LoCHO diets have been reported to reduce fat mass including truncal fat over long term in many studies [11,21,26,29]. Finally, a recent study showed that effectiveness of low carbohydrate diets was more visible in a group that was insulin-resistant [30].

## Low carbohydrate diet and hypertension

Hypertension is a common co-morbidity in type 2 diabetes affecting 20–60% of the diabetic population[74] and contributes significantly to CVD risk. Hypertension is a major predictor of increased macrovascular and microvascular complications of diabetes [17,52,53,75]. Hypertension in diabetes is usually a component of metabolic syndrome and is related to carotid wall atherosclerotic lesions and angina [17]. A number of studies in animals [76] and one in humans [77] have linked sugar intake with hypertension. Direct correlation between plasma insulin levels and blood pressure levels has been demonstrated and there is evidence to suggest a causal relationship between insulin resistance with resultant hyperinsulinemia and hypertension [17]. The proposed mechanisms include renal sodium retention, vascular smooth muscle proliferation, sympathetic stimulation and vascular hyperreactivity [17].

The role of macronutrient composition of diet on blood pressure has not been adequately studied, though any dietary intervention effective for improving insulin resistance should also have beneficial effects on hypertension. The relationship between hypertension and weight loss is well documented [13,74] and weight loss of 1 kilogram has been reported to decrease mean arterial blood pressure by approximately 1 mm Hg. Low carbohydrate diets have been reported to lower blood pressure by causing weight loss and improving the insulin sensitivity, though the magnitude of effect on blood pressure has been small (1–10 mm Hg) in most studies [8,10,29] and comparable to that seen with low fat diet.

## Low carbohydrate diet and inflammation

Insulin resistance is the predominant mechanism associated with type 2 diabetes and is also central to the pathogenesis of metabolic syndrome [17]. Abnormal levels of inflammatory markers such as C-reactive protein and prothrombotic markers like plasminogen activator inhibitor-1 (PAI-1) have been reported in insulin resistant subjects [17] and may contribute to the increased CVD events in this population in combination with dyslipidemia and hypertension [17]. High carbohydrate diets, by increasing the insulin secretion, may worsen insulin resistance in diabetic patients and increase the inflammatory and prothrombotic tendencies in this patient population. The effect of low carbohydrate diet on various inflammatory and pro-coagulant markers is not well studied although these markers have been shown to improve with weight loss in general [13,78-80]. Therefore, any diet that causes effective weight loss should be able to cause a decrease in these inflammatory markers and such decreases in CRP [32,70] and PAI-1 levels [29] have been demonstrated with low carbohydrate diet. However, these data are limited, and long term studies are needed to confirm these findings and to determine the impact of these parameters on the CVD end points with low carbohydrate diet.

## The case for a low carbohydrate diet

Despite the growing popularity of LoCHO diets and emerging evidence for its effectiveness, there is reluctance among physicians to prescribe it, even in high risk populations who have failed to benefit from traditional low fat diets. The possible explanation is that although carbohydrate restriction can be implemented in any number of ways, it is generally identified with the popular Atkins diet for which health organizations have great hostility, focusing on a literal interpretation of permission for unlimited fat intake. Health organizations have been slow to adapt carbohydrate restricted diets even in the face clear evidence for the adverse effects of high carbohydrate intake exacerbating the metabolic abnormalities in diabetes and insulin resistant states. Similarly, we have previously pointed out the general tendency to downplay the beneficial effects of LoCHO diets by individual researchers [81]. Weight loss associated with LoCHO diets has been continually attributed to alteration in body water [82,83]. Numerous studies have shown that, although initial alteration in body water with carbohydrate restriction is possible, the new equilibrium state is achieved in 2–3 weeks, followed by active loss of fat mass [8,20,21,26,29,73]. In fact, a recent study [9] showed mean energy intake decreased from 3111 kcal/day to 2164 kcal/day on a low carbohydrate diet with mean energy deficit of 1027 kcal/day. The weight loss was proportional to mean energy deficit and was explained by loss of fat mass, not water loss.

Another concern that has been voiced is unlimited fat intake as part of low carbohydrate diet may cause weight gain and obesity over the long term. [34,47]. Again, this is not substantiated with evidence and the objection is not valid for the many reasons. Firstly, there is evidence that type of fat is much more important than total fat intake [84]. Whereas saturated fats have been linked with increased CVD risk, the use MUFA and PUFA have been inversely associated with CVD risk [84]. Therefore, if carbohydrate restriction is used with increase in unsaturated fats, the benefits may be even more and indeed, improvement in glycemic control, insulin sensitivity and dyslipidemia including reduction in LDL cholesterol has been demonstrated with such dietary intervention in several clinical and metabolic studies[3,41,85].

Another major reason for lack of enthusiasm for LoCHO diet is misinterpretation of data provided by studies up to 1 year duration. The general view is that the LoCHO diet are not more effective than low fat high carbohydrate diet in terms of weight loss in studies up to 1 year duration despite impressive short term weight loss [8,27,32]. However, as we mentioned earlier, considering that the data is already biased in favor of low fat diet, the lack of significance between the diet groups at 12 months still proves the superiority of LoCHO diet as it was able to achieve similar or better weight loss despite less than desired carbohydrate restriction. Again, even a marginal decrease in carbohydrate intake may be beneficial in terms of weight loss and lipid benefits. The overall dietary compliance was a major problem in all these studies but was generally comparable between the two diet groups. A lot has been said about nutritional inadequacy due to mineral, electrolytes and vitamin deficiencies and the adverse health effects of LoCHO diets on renal and skeletal mass [34]. On the other hand, there is evidence that increasing sugar intake adds empty calories by displacement of whole foods and has been associated with a linear decrease in intake of many essential nutrients as demonstrated by Bogalusa Heart Study [86]. Since carbohydrate restriction may limit some micronutrient and fiber intake, the popular versions of LoCHO diets recommend mandatory intake of multivitamins including calcium, fish oil and fiber supplements. The renal and skeletal effects remain theoretical concerns not substantiated with evidence. Studies up to 1 year have not shown any adverse effects on renal function or skeletal mass. Also, adequate intake of calcium and vitamin D supplements as routinely recommended with LoCHO diet should help preserve skeletal mass along with prescription of increased physical activity which should be offered to all irrespective of the dietary macronutrient composition. There have been some reports of increase blood urea nitrogen without any decrease in glomerular filtration which are related to increase in protein intake and do not represent renal insufficiency. A theoretically increased risk of renal stones has been claimed with LoCHO diet but again, there is no evidence for such claims and nothing that adequate hydration could not correct. However, in the absence of definite evidence, recommendations must be based on professional judgment. Although proponents of LoCHO diets recommend *ad libitum *fat intake, we do not endorse this and instead favor use of MUFA and PUFA which have been demonstrated in various studies to reduce the risk of CVD [3,4,41,84]. A final irony is the report that physicians frequently choose LoCHO diets for themselves while recommending low fat for their patients[87].

## Where we stand

Based on our examination of current evidence, we find concerns about LoCHO diets to be unsubstantiated and we see no problem in recommending them, at least as a means of caloric reduction. Of course, reducing calories by removing fat is universally agreed on as beneficial but the real question is which should be the priority. We believe from the evidence presented here that replacing fat with carbohydrate is deleterious and caloric restriction should be carried out by removing carbohydrate in preference to removing fat. Although calorie counting is not recommended by various popular LoCHO diets, we routinely remind our patients to avoid excess calorie intake. Also, because restriction of carbohydrates may limit intake of certain vitamins and minerals, supplementation with multivitamin supplements including calcium is a usual recommendation with LoCHO diets. Since high fiber intake has been inversely associated with CVD [37,88], patients should be encouraged to increase fiber intake and should receive fiber supplements if necessary.

As for VLCKD where carbohydrate restriction is targeted to 20–30 gm/day at least for two weeks, we consider this an extreme change for most people and therefore we would not recommend it without substantial evidence from clinical trials is provided as to the benefits of this extreme dietary intervention.

## Low Carbohydrate diet and the current guidelines for diabetes management

Traditionally, a low fat high carbohydrate diet containing 55–70 % carbohydrates, 15–20% proteins and 20–30% fats has been recommended by various health organizations [33-35] for subjects at high risk of CVD including those with diabetes and metabolic syndrome. Despite accumulating evidence suggesting deleterious effects of high carbohydrate diet and potential benefits of carbohydrate restriction, LoCHO diet have met with increased resistance and have not been accorded its deserved place in various treatment guidelines.

The current position statement of the American Diabetes Association (ADA), however, recognizes the importance of amount of dietary carbohydrates: "*With regard to the glycemic effects of carbohydrates, the total amount of carbohydrate in meals or snacks is more important than the source or the type"*. This organization also agrees with role of carbohydrate restriction as stated "*In weight maintaining diets for type 2 diabetes, replacing carbohydrates with monounsaturated fats reduces post prandial glycemia and triglyceridemia" *and recommends that *"carbohydrates and monounsaturated fat together should provide 60–70% of the energy intake and the relative contributions of carbohydrate and monounsaturated fats to energy intake should be individualized*". This can be considered as a nod of approval for carbohydrate restriction though no specific recommendation has been made. Furthermore, the ADA's recommendation of individualization according to patient's risk may provide the much needed flexibility for adjusting the carbohydrate content according to the patient's requirement [47]. This gradual adoption of carbohydrate restriction is also reflected by a recent statement from ADA limiting dietary carbohydrate intake to provide 45–65% of the calories [47] which is less than previously recommended.

## Conclusion

Low carbohydrate diet compares more favorably, at least over the short term, to traditional low fat for improving glycemic control, insulin sensitivity and dyslipidemia of diabetes with reduction in triglycerides, increase in HDL cholesterol and modification of LDL to less atherogenic form. The need of the hour is to accept the benefits of carbohydrate restriction with reservation and to establish guidelines for its use, especially emphasizing use of mono and polyunsaturated fats as the way to achieve caloric balance since these have been inversely linked with CVD risk in various studies. In the mean time, clinical trials need to be conducted using graded levels of carbohydrate restriction and fat intake, with special emphasis on unsaturated fats, to examine their effects of on weight loss, glycemic control, insulin resistance and CVD risk. This is to resolve the present controversy about optimal dietary option for patients with diabetes.

## Competing interests

The author(s) declare that they have no competing interests.

## Authors' contributions

SA conducted literature search, prepared the manuscript and helped in presentation of final draft, SIM conceived the idea, organized the contents and helped in the preparation and presentation of final manuscript.
